# Repression of genes involved in melanocyte differentiation in uveal melanoma

**Published:** 2012-07-04

**Authors:** Marjorie-Allison Bergeron, Sophie Champagne, Manon Gaudreault, Alexandre Deschambeault, Solange Landreville

**Affiliations:** LOEX/CUO-recherche, Centre hospitalier *affilié* universitaire de Québec, Hôpital du Saint-Sacrement, Département d’ophtalmologie, Faculté de médecine, Université Laval, Québec, Québec, Canada

## Abstract

**Purpose:**

Uveal melanoma (UM) has been the subject of intense interest due to its distinctive metastatic pattern, which involves hematogenous dissemination of cancerous cells toward the liver in 50% of patients. To search for new UM prognostic markers, the Suppressive Subtractive Hybridization (SSH) technique was used to isolate genes that are differentially expressed between UM primary tumors and normal uveal melanocytes (UVM).

**Methods:**

A subtracted cDNA library was prepared using cDNA from uncultured UM primary tumors and UVM. The expression level of selected genes was further validated by cDNA microarray, semi-quantitative reverse transcription polymerase chain reaction (RT-PCR), and immunofluorescence analyses.

**Results:**

One hundred-fifteen genes were identified using the SSH technique. Microarray analyses comparing the gene expression profiles of UM primary tumors to UVM validated a significant differential expression for 48% of these genes. The expression pattern of selected genes was then analyzed by semi-quantitative RT–PCR and was found to be consistent with the SSH and cDNA microarray findings. A down-regulation of genes associated with melanocyte differentiation was confirmed in UM primary tumors. Presence of undifferentiated cells in the UM was demonstrated by the expression of stem cell markers ATP-binding cassette sub-family G member 2 (ABCG2) and octamer-binding protein 4 (OCT4).

**Conclusions:**

We demonstrated that the SSH technique is efficient to detect differentially expressed genes between UM and UVM. The genes identified in this study represent valuable candidates for further functional analysis in UM and should be informative in studying the biology of this tumor. In addition, deregulation of the melanocyte differentiation pathway revealed the presence of UM cells exhibiting a stem cell-like phenotype.

## Introduction

Among all melanoma cases reported in North America, approximately 5% arise from the eye [1]. Uveal melanoma (UM) is the most frequent intraocular tumor in adult population [2]. Despite the primary tumor being treated successfully by radiation or enucleation, the mortality rate remains high among patients who develop metastatic disease. Indeed, more than 50% of patients will be diagnosed with metastatic cancer within a few years following the treatment of the primary tumor [3]. Those secondary tumors can spread to many organs, including liver (93%), lungs (24%), and bones (16%) [4]. When the liver is involved, which occurs in most of the cases, there is little chance of survival for the patient; estimated median survival after detection of metastatic disease is about 6 months [4].

Clinical features of the primary tumor can help predict prognosis of patients, such as the size of the tumor and its location: large tumors involving ciliary body have the worst prognosis [5,6]. Examination of enucleated eyes allowed the identification of several histopathological prognostic factors, such as epithelioid cell morphology, extent of mitotic activity, as well as presence of microvascular patterns, tumor-infiltrating lymphocytes and extrascleral extension [5,6]. In addition, cytogenetic modifications on chromosomes 1, 3, 6, and 8 are frequently observed in UM, and monosomy 3 and 8p loss are strongly associated to metastatic death [2,7]. Since chromosome 6p gain (non-metastasizing tumors) and monosomy chromosome 3 (metastasizing tumors) are mutually exclusive, these events can predict metastatic potential of UM tumors [7,8].

Recent studies allowed the identification of molecular and genetic markers of UM. It is now possible to classify UM cases in two distinct groups according to their gene expression profiles: class 1 tumors with a low-risk, and class 2 tumors with a high-risk of metastasis [9]. More than 80% of UM tumors have mutations in guanine nucleotide binding protein q polypeptide (*GNAQ*) and guanine nucleotide binding protein alpha 11 (*GNA11*) G-proteins that lead to constitutive activation of the mitogen-activated protein kinase (MAPK) pathway [10-12]. These mutations are also found in benign nevi, suggesting they are initiating events in UM progression [10]. Furthermore, inactivating somatic mutations were identified in BRCA1 associated protein-1 (*BAP1*), which occur in 84% of metastasizing tumors, implying that BAP1 inactivation is a key event in metastasis spreading [13].

In spite of these new findings, metastases remain fatal in absence of their early detection. Better insight into UM neoplastic and metastatic mechanisms is thus of utmost importance. Finding genes involved in these processes shall therefore provide useful information. The suppressive subtractive hybridization (SSH) approach systematically compares cDNAs from two different tissue types and preferentially depletes sequences common in both, thereby enriching sequences specific for one tissue type [14]. Identification of discriminating low-expression genes by microarray is challenging due to the detection sensitivity limitations for rare genes and signal saturation for highly abundant genes. A subtractive library was thus prepared to identify differentially expressed genes in UM primary tumors compared to normal uveal melanocytes (UVM).

## Methods

This study followed the tenets of the Declaration of Helsinki and was approved by our institutional human experimentation committee. Written informed consent was obtained from the enucleated subjects.

### Tumor samples and tissue culture

UVM were grown from normal human donor eyes (provided by the Banque d’Yeux du CUO, Québec, QC, Canada). Isolation and culture of low-passage UVM (8 donors; median 58-year-old) were performed as described previously [15-17]. Samples of UM primary tumors were collected at the time of enucleation and the biopsies were either immediately stored at −80 °C in Tri-Reagent (Sigma-Aldrich, Oakville, ON, Canada) for RNA extraction, embedded in Optimal Cutting Temperature compound (OCT; Somagen, Edmonton, AB, Canada) and frozen at −80 °C for indirect immunofluorescence, or grown in tissue culture for less than 5 passages for protein extraction as described previously [17]. Clinicopathological characteristics and survival data of UM cases used in the present study are compiled in Table 1 (14 patients; median 56-year-old). Tumors were classified according to a modification of the Callender’s classification [18]. All normal donors and UM patients were Caucasians.

**Table 1:** Clinicopathological characteristics and survival data of uveal melanoma cases investigated.

**Age, Sex**	Tumor location/Size*	Tumor pigmentation**	Last status***	Follow-up (months)****	**Pathology**
67, M	Choroid/med	0	ANM	100	spindle
73, M	Choroid and ciliary body/med	1	DOO	18	mixed
45, M	Choroid/med	1	ANM	94	spindle
57, M	Choroid/med	2	ANM	93	spindle
57, M	Choroid/med	0	DOM	47	mixed
64, F	Choroid and ciliary body/lg	2	DOM	17	mixed
63, F	Choroid and ciliary body/lg	2	DOO	3	mixed
46, F	Choroid/lg	1	ANM	79	spindle
64, F	Choroid and ciliary body/lg	2	DOM	17	mixed
51, M	Choroid and ciliary body/lg	2	ANM	48	epithelioid
44, M	Choroid/med	1	ANM	40	epithelioid
53, M	Choroid and ciliary body/lg	1	ANM	12	epithelioid
55, M	Choroid/med	1	ANM	12	spindle
47, F	Choroid and ciliary body/lg	1	DOM	58	mixed

*Size classification: sm, small; med, medium; lg, large. **Tumor pigmentation: 0, not pigmented; 1, moderately pigmented; 2, highly pigmented. ***Last status: ANM, alive no metastasis; DOM, dead of metastasis; DOO, dead of other. ****Follow-up: period from enucleation until patient death or last visit.

### Suppressive subtractive hybridization (SSH), cloning, differential screening, sequencing and analysis of the subtracted cDNAs

Total RNA from UVM (pool of 8 donors) and from uncultured UM primary tumors (pool of 9 patients) was extracted with the RNeasy kit (Qiagen, Mississauga, ON, Canada) or Tri-Reagent (Sigma-Aldrich), and mRNA was isolated with the Oligotex mRNA kit (Qiagen). cDNA was synthesized using the SMART PCR cDNA Synthesis kit (Clontech Laboratories, Mountain View, CA) and SSH was then performed between UVM and UM primary tumors (subtracted cDNA library) using the PCR-Select cDNA Subtraction kit (Clontech Laboratories) according to the procedure described by Diatchenko et al. [14]. UVM cDNA was used as tester and UM primary tumors cDNA as driver, which generates down-regulated genes in UM. To evaluate the efficiency of the cDNA subtraction, the expression level of *ACTB* (housekeeping gene; see primer sequences in Table 2) was monitored by reverse transcription polymerase chain reaction (RT–PCR) in subtracted cDNA and unsubtracted UVM cDNA. Aliquots of subtracted and unsubtracted cDNAs were taken from each reaction after 18, 23, 28, and 33 cycles and compared by agarose gel electrophoresis. Cloning, differential screening, sequencing and analysis of the subtracted cDNAs were performed as described previously [17]. The PCR products of the SSH library were purified (NucleoSpin Extract kit; Clontech Laboratories), and then inserted into the T/A cloning vector pGEM-T Easy (Promega, Madison, WI). Individual transformants carrying subtracted cDNA fragments were isolated from white colonies and used for differential screening (PCR-Select Differential Screening kit; Clontech Laboratories) to eliminate false positives, according to the manufacturer’s instructions. PCR fragments of the positive clones were isolated with the QIAquick PCR Purification kit (Qiagen), and then sequenced with an automated DNA sequencer (ABI Prism model 3900; Applied Biosystems, Foster City, CA) using Nested PCR Primers 1 and 2R (Clontech Laboratories). DNA sequencing of positive clones was performed by the Plateforme de séquençage et de génotypage des génomes at Université Laval (Québec, QC, Canada). The inserted sequences were examined for similarities to human genes with the NCBI BLAST program. A sequence was considered significant to a database entry when an aligned region was more than 95% identical over the entire cDNA length.

**Table 2:** Sequence of forward and reverse primers used for PCR amplification.

**Gene**	**Forward Primer (5′-3′)**	**Reverse Primer (5′-3′)**	**Expected PCR Product Size (bp)**
*ACTB*	TGTCCACCTTCCAGCAGATGT	CACTCCCAGGGAGACCAAAA	609
*ADAM10*	AGGGAACGAGCAAGGGAAGG	GCCACCACGAGTCTGGATGA	660
*CALU*	TGATGCCTTCTTGGGTGCTG	CCTGGCTTCTGCCTCTGCAT	667
*CTNNB1*	TGGCCATGGAACCAGACAGA	GGTCCCAGCGGTACAACGAG	617
*DCT*	TGGTGAGAAGCGCTACCCTCAT	AGCCGGCAAAGTTTCCTGTG	601
*EDNRB*	CCAACATGTGGCCCAGCCTA	TGAGGTGGGGTTGGAGGAAA	231
*PGCP*	TCCCTGTGCTCTGGGAAAGC	GGCACGCCATCCTGGTATTC	635
*RAB27A*	AACAAGCGGTTCTCTACCCTGT	TCCACACACCGTTCCATTCG	617
*TRPM1*	CAGGGTGGCGGATATTCCAA	CGCTGCCATCACAAATCACC	664
*TYRP1*	ACCGCTGTGGCTCATCATCA	TCCCCGTTGCAAAATTCCAG	603

### Microarray gene expression profiling

Gene expression profiling was performed by the Plateforme Agilent of the LOEX/CUO-recherche (Québec, QC, Canada) using a SurePrint G3 Human GE 8x60K array slide (60,000 probes; Agilent Technologies Canada, Mississauga, ON, Canada) [19]. The analysis of the relative expression level of mRNAs and of the hierarchical clustering of differentially expressed genes was performed using ArrayStar v4.1 software (DNASTAR, Madison, WI). UVM gene expression profile was compared to those of UM primary tumors with good prognosis (5-hydroxytryptamine (serotonin) receptor 2B (*HTR2B*)^-^, spindle cells, tumor did not invade the ciliary body) and bad prognosis (*HTR2B^+^*, epithelioid cells, tumor invaded the ciliary body) using established prognostic factors [9,20].

### Semi-quantitative RT–PCR

Reverse transcription using total RNA from UVM (pool of 8 donors) and uncultured UM primary tumors (pool of 9 patients) was performed using random hexamer primers following manufacturer’s protocol for synthesis of first strand cDNA (MBI Fermentas, Burlington, ON, Canada). Gene specific primers (Table 2) were designed using the NCBI GenBank database and synthesized by the Service de synthèse d’ADN du CHUQ (Québec, QC, Canada). Semi-quantitative RT-PCR was performed using the QuantumRNA 18S Internal standards protocol (Ambion, Austin, TX) according to the manufacturer’s instructions [17]. The cycle parameters were 94 °C for 30 s, 60 °C for 30 s, and 72 °C for 30 s, and the number of cycles was chosen in the linear range for each gene. The 18S primers (Ambion) gave a PCR product of 489 bp. PCR band density and ratio were determined using ImageJ as described previously [21]. The intensity of each PCR band was divided by the length of the corresponding PCR product and normalized with the intensity of the corresponding 18S RNA band. Ratios were calculated by dividing normalized values of UM by UVM.

### Immunofluorescence

Staining was performed on acetone-fixed cryosections (5 μm). Sections were blocked in 5% normal goat serum, and then incubated for 45 min at room temperature with optimal dilution of antibodies directed against ATP-binding cassette sub-family G member 2 (ABCG2; mouse, 10 µg/ml; Cedarlane, Burlington, ON, Canada) and octamer-binding protein 4 (OCT4; mouse, 10 µg/ml; Cedarlane). The sections were then incubated for 30 min with a goat anti-mouse Alexa 594-conjugated secondary antibody (Molecular Probes; Life Technologies, Burlington, ON, Canada). Cell nuclei were counterstained with the Hoechst 33258 reagent (Sigma-Aldrich). Samples were then observed with an epifluorescence microscope (Eclipse E600; Nikon, Montréal, QC, Canada) and photographed with a CCD camera (Sensys; Roper Scientific, Trenton, NJ). For the negative control, the primary antibody was replaced by mouse IgG (10 µg/ml; Millipore, Billerica, MA).

## Results

### Evaluation of subtraction efficiency

**Figure 1:**
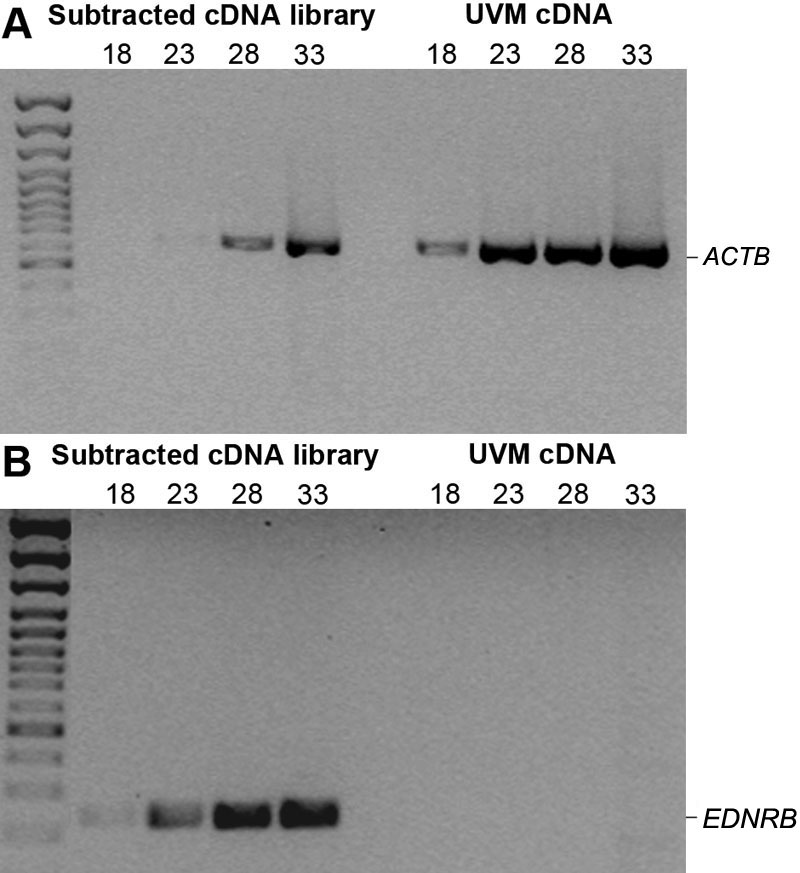
Evaluation of the subtraction efficiency by PCR using the housekeeping gene *ACTB* and the melanocyte marker *EDNRB*. **A**: Depletion of *ACTB* expression (609 bp) in the subtracted cDNA library compared to unsubtracted UVM cDNA. **B**: Enrichment of *EDNRB* expression (231 bp) in the subtracted cDNA library compared to unsubtracted UVM cDNA. Samples were taken after 18, 23, 28, and 33 PCR cycles and separated by electrophoresis.

Successful SSH should decrease housekeeping gene transcripts abundance and enrich tissue-specific gene transcripts [23]. A depletion of *ACTB* mRNA expression can be observed in the subtracted cDNA library compared to unsubtracted UVM cDNA. Indeed, the subtracted cDNA library requires a higher number of amplification cycles for *ACTB* to be detected (faint amplicon at 23 cycles), indicating that *ACTB* is preferentially depleted in the subtracted cDNA library (Figure 1A). As a positive control for the enrichment of differentially expressed genes, a melanocyte marker endothelin receptor type B (*EDNRB*) was amplified. *EDNRB* amplicon can be observed after 18 cycles in the subtracted cDNA library compared to 33 cycles for the unsubtracted UVM cDNA where the band is barely visible (Figure 1B). These data demonstrate successful subtraction, with significant depletion of *ACTB* abundance and enrichment of *EDNRB*.

### Comparison of mRNA profiles between UVM and UM primary tumors using SSH and microarray

**Figure 2:**
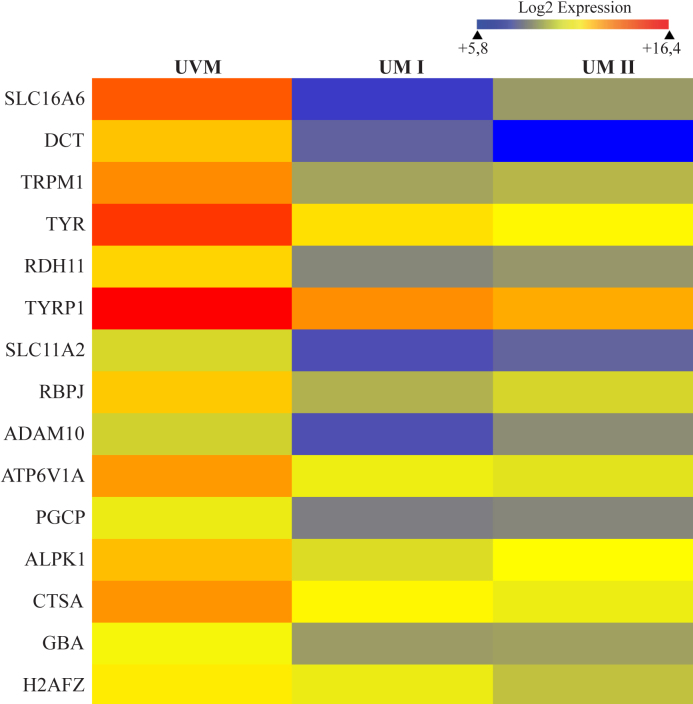
Hierarchical clustering of the top 15 differentially expressed genes identified by the SSH technique and validated by microarray. UVM gene expression profile was compared to those of UM primary tumors with good (UM I) or bad prognosis (UM II). The color scale above the heat map diagram illustrates the relative expression level of mRNAs (log2 expression): red color represents a high expression level; blue color represents a low expression level.

Analysis of 432 colonies obtained with the subtracted cDNA library (genes down-regulated in UM) showed that 175 positive clones corresponded to 115 known genes when taking the redundancy into account. Comparison by microarray of the UVM transcriptome with those of two UM primary tumors (with good or bad prognosis) confirmed a significant down-regulation of 48% of these genes (Table 3; fold-change >1.5). The most represented biologic processes were associated to melanocyte differentiation, development, and energetic metabolism. The most redundant genes in the subtracted cDNA library were calumenin (*CALU*), catenin beta 1 (*CTNNB1*), dopachrome tautomerase (*DCT*), sorting nexin family member 27 (*SNX27*), tyrosinase-related protein 1 (*TYRP1*), and vacuolar protein sorting 35 (*VPS35*). Monosomy 3 occurs mostly in metastasizing tumors and 8 of the repressed genes are mapped on chromosome 3: ATPase H+ transporting lysosomal 70kDa V1 subunit A (*ATP6V1A*), *CTNNB1*, coatomer protein complex subunit beta 2 (*COPB2*), microphthalmia-associated transcription factor (*MITF*), signal peptidase complex subunit 1 homolog (*SPCS1*), transferrin receptor (*TFRC*), translocase of inner mitochondrial membrane domain containing 1 (*TIMMDC1*), and ubiquitin specific peptidase 4 (*USP4*) (Table 3) [2]. Hierarchical clustering of the 55 genes identified in the SSH library and validated by microarray allowed to determine that solute carrier family 16 member 6 (*SLC16A6*) was the most repressed gene in UM with fold-change values of −182 (UM I) and −47 (UM II), respectively (Table 3). The top 15 down-regulated genes are represented in a heat map diagram in Figure 2.

**Table 3:** SSH differentially expressed genes validated by cDNA microarray analysis (fold-change >-1.5).

**Genes**	**Chromosomal location**	**GenBank accession#**	**Biological process**	**SSH redundancy**	**Microarray fold-change UMI/UVM**	**Microarray fold-change UMII/UVM**
ADAM metallopeptidase domain 10 (ADAM10)	15q22	NM_001110	NOTCH signaling	1	−6.37	−2.68
Adenosylhomocysteinase-like 1 (AHCYL1)	1p13.2	NM_006621	Ion transport	1	−1.51	−1.10
Alpha-kinase 1 (ALPK1)	4q25	NM_025144	Protein phosphorylation	1	−4.19	−2.46
ATPase H^+^ transporting lysosomal 70 kDa V1 subunit A (ATP6V1A)	3q13.31	NM_001690	ATP hydrolysis	1	−5.37	−6.27
BCL2-associated transcription factor 1 (BCLAF1)	6q23	NM_014739	Apoptosis	1	−4.83	−1.33
Calumenin (CALU)	7q32.1	NM_001219	Calcium binding	5	−2.01	−1.51
Casein kinase 1 gamma 1 (CSNK1G1)	15q22.31	NM_022048	WNT signaling	1	−1.50	−1.51
Casein kinase 2 alpha prime polypeptide (CSNK2A2)	16q21	NM_001896	WNT signaling	1	−1.12	−1.75
Catenin (cadherin-associated protein) beta 1 (CTNNB1)	3p21	NM_001904	WNT signaling	4	−1.38	−2.33
Cathepsin A (CTSA)	20q13.1	NM_000308	Protein transport	1	−4.07	−6.00
CCR4-NOT transcription complex subunit 2 (CNOT2)	12q15	NM_014515	RNA splicing	2	−2.12	−1.67
Chromosome 7 open reading frame 64 (C7orf64)	7q21.2	NM_032120	Nucleotide metabolism	1	−2.10	−2.25
Coatomer protein complex subunit beta 2 (COPB2)	3q23	NM_004766	Protein transport	1	−1.30	−1.66
Dolichyl-phosphate N-acetylglucosaminephosphotransferase 1 (DPAGT1)	11q23.3	NM_001382	Protein glycosylation	1	−1.56	−1.41
Dopachrome tautomerase (DCT)	13q32	NM_001922	Melanocyte differentiation	4	−22.30	−89.81
Drosha RNase type III (DROSHA)	5p13.3	NM_013235	Gene silencing	1	−2.08	1.10
Eukaryotic translation initiation factor 4 gamma 3 (EIF4G3)	1p36.12	NM_003760	Translation	1	−5.41	−1.43
Glucosidase beta acid (GBA)	1q21	NM_000157	Glycolipid metabolism	1	−3.67	−3.40
H2A histone family member Z (H2AFZ)	4q24	NM_002106	Embryonic development	2	−1.72	−3.18
Integrin beta 8 (ITGB8)	7p21.1	NM_002214	Cell adhesion	1	−1.90	−1.33
Isoleucine-tRNA synthetase (IARS)	9q21	NM_013417	Translation	1	−1.66	1.17
Karyopherin alpha 2 (KPNA2)	17q24.2	NM_002266	Cell cycle	1	−1.15	−1.50
Lactate dehydrogenase B (LDHB)	12p12.1	NM_002300	Glycolysis	1	−1.68	1.97
Leucyl-tRNA synthetase (LARS)	5q32	NM_020117	ATP hydrolysis	1	−2.57	−1.08
Lysosomal trafficking regulator (LYST)	1q42.3	NM_000081	Pigmentation	1	−5.59	−5.15
Malectin (MLEC)	12q24.31	NM_014730	Protein glycosylation	1	−1.61	−1.36
RAB27A member RAS oncogene family (RAB27A)	15q21.3	NM_004580	Melanocyte differentiation	3	−2.64	−1.76
Microophthalmia-associated transcription factor (MITF)	3p14.1	NM_198159	Melanocyte differentiation	1	−2.91	−2.11
NHS-like 1 (NHSL1)	6q23.3	NM_020464	Unknown	1	−1.95	−2.35
Nucleoporin 93 kDa (NUP93)	16q13	NM_014669	Glycolysis	1	−2.18	−2.16
Plasma glutamate carboxy-peptidase (PGCP)	8q22.2	NM_016134	Proteolysis	2	−4.78	−4.30
Prosaposin (PSAP)	10q21	NM_002778	Lipid metabolism	1	−1.39	−1.87
Prostaglandin reductase 1 (PTGR1)	9q31.3	NM_012212	LTB4 metabolism	1	−2.22	−1.17
Protein kinase D3 (PRKD3)	2p21	NM_005813	PKC signaling	1	−3.59	−2.69
Radixin (RDX)	11q23	NM_002906	Microvillus assembly	1	−1.71	−1.07
Ras-like without CAAX 1 (RIT1)	1q22	NM_006912	NGFR signaling	1	−1.27	−1.67
Recombination signal binding protein for immunoglobulin kappa J region (RBPJ)	4p15.2	NM_005349	NOTCH signaling	2	−6.66	−3.94
Retinol dehydrogenase 11 (RDH11)	14q24.1	NM_016026	Retinoid metabolism	1	−10.21	−8.28
Serine carboxy-peptidase 1 (SCPEP1)	17q22	NM_021626	Retinoic acid metabolism	1	−2.52	−3.29
Signal peptidase complex subunit 1 (SPCS1)	3p21.1	NM_014041	Insulin secretion	1	1.68	−1.50
Solute carrier family 11 member 2 (SLC11A2)	12q13	NM_000617	Iron absorption	1	−7.21	−5.27
Solute carrier family 16 member 6 (SLC16A6)	17q24.2	NM_004694	Glycolysis	2	−181.62	−46.58
Solute carrier family 25 member 5 (SLC25A5)	Xq24	NM_001152	Cell respiration	1	−1.37	−1.61
Sorting nexin family member 27 (SNX27)	1q21.3	NM_030918	Protein transport	5	−1.61	−1.09
Sperm associated antigen 5 (SPAG5)	17q11.2	NM_006461	Cell cycle	1	−1.80	−1.14
Subunit of the oligosaccharyl-transferase complex homolog A (STT3A)	11q23.3	NM_152713	Protein glycosylation	2	−2.03	−1.68
Transaldolase 1 (TALDO1)	11p15.5	NM_006755	Cell respiration	1	−1.23	−1.87
Transcription elongation factor B (SIII) polypeptide 1 (TCEB1)	8q21.11	NM_005648	Transcription	1	−1.65	2.43
Transferrin receptor (TFRC)	3q29	NM_003234	Response to hypoxia	1	−4.40	−4.12
Transient receptor potential cation channel subfamily M member 1 (TRPM1)	15q13.3	NM_002420	Melanocyte differentiation	1	−19.16	−14.62
Translocase of inner mitochondrial membrane domain containing 1 (TIMMDC1)	3q13.33	NM_016589	Protein transport	1	−1.51	−2.32
Tyrosinase (TYR)	11q14.3	NM_000372	Melanocyte differentiation	1	−11.23	−15.84
Tyrosinase-related protein 1 (TYRP1)	9p23	NM_000550	Melanocyte differentiation	10	−7.67	−11.58
Ubiquitin specific peptidase 4 (USP4)	3p21.3	NM_003363	Protein deubiquitination	1	−2.61	−3.02
Vacuolar protein sorting 35 (VPS35)	16q12	NM_018206	Protein transport	4	−1.18	−1.72

### Validation of down-regulated genes by semi-quantitative RT–PCR

**Figure 3:**
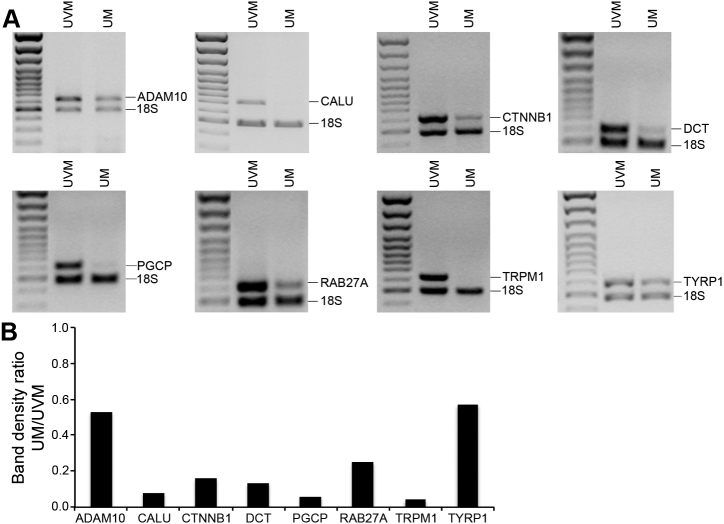
Validation of differentially expressed genes identified by the subtracted cDNA library and confirmed by microarray gene expression profiling. **A**: The mRNA expression level of selected genes down-regulated in UM (*ADAM10*, *CALU*, *CTNNB1*, *DCT*, *PGCP*, *RAB27A*, *TRPM1*, and *TYRP1*) was measured by semi-quantitative RT–PCR in pools of RNA from UVM and uncultured UM primary tumors (UM). The 18S RNA was used as an internal control of amplification (489 bp). **B**: Band density ratio calculated from A panels. 18S RNA band density was used for internal normalization, and ratio was calculated by dividing normalized values of UM by UVM.

Semi-quantitative RT–PCR analyses were performed to compare the expression level of ADAM metallopeptidase domain 10 (*ADAM10*), *CALU*, *CTNNB1*, *DCT*, plasma glutamate carboxypeptidase (*PGCP*), RAB27A member RAS oncogene family (*RAB27A*), transient receptor potential cation channel subfamily M member 1 (*TRPM1*), and *TYRP1* between UVM and UM primary tumors (Figure 3A). These genes were chosen for their redundancy in the SSH library and/or their repression by microarray. These analyses showed similar expression patterns as those revealed by SSH and microarray data. Indeed, band density ratio UM/UVM indicated a decrease of PCR products in UM for all genes after normalization with the 18S (Figure 3B).

### Expression of stem cell markers in UM

**Figure 4:**
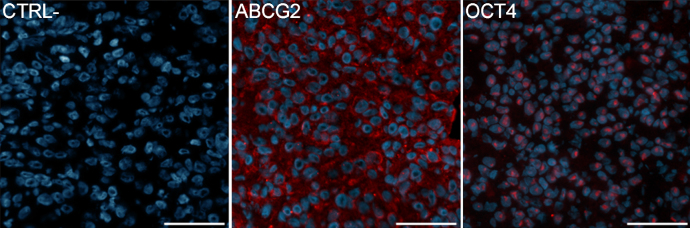
Expression of stem cell markers in UM. ABCG2 (middle panel) and OCT4 (right panel) protein expression was assessed by indirect immunofluorescence in a mixed UM primary tumor. Positive staining appears in red. Nuclei were counterstained using Hoechst (in blue). No positive staining was observed in the negative control section using mouse IgG (left panel). Scale bars, 50 μm.

In many cancers, tumor cells demonstrate an undifferentiated phenotype. The down-regulation of many genes involved in melanocyte development and differentiation in this study and in a previous subtracted cDNA library [17], prompted us to verify the expression of stem cell markers in UM. Positive staining with ABCG2 and OCT4 was demonstrated on a mixed UM primary tumor (Figure 4). ABCG2 expression was both membranous and cytoplasmic (Figure 4, left panel). The presence of functional ABCG2 transporters in UM cells was previously demonstrated indirectly using the side population assay [16]. Cytoplasmic accumulation of ABCG2 has been described in poorly differentiated tumors with defective phosphoinositide-3-kinase (PI3K) signaling pathway [24]. The expression of the transcription factor OCT4 was localized in the nucleus (Figure 4, right panel). Both stem cell markers were detected in majority of cells that constitute the tumor. Negligible background was observed for the negative control.

## Discussion

The SSH technique is a powerful approach to enrich tissue- or cancer-specific genes [14]. To identify new markers with a prognostic value, a subtracted cDNA library was prepared to isolate genes that are repressed in UM primary tumors compared to UVM. The sequencing of the resulting subtracted cDNAs and further validation by microarray has resulted in the identification of 55 genes. The efficiency of the subtraction was shown by depletion of the highly and ubiquitously expressed gene *ACTB*. Furthermore, the presence of other known melanocyte-specific genes, such as *DCT* and *TYRP1*, at high frequency in the subtracted cDNA library further demonstrates that the subtraction process worked effectively. SSH was previously used to identify genes that are differentially expressed in UM [25-27]. An extensive comparison of our results with these data was not possible to perform since only 4 genes were reported in these studies [25-27]. Also, the type of cells or tissues used for these libraries were different: the subtracted cDNA library published by the group of Polans [26,27] was prepared using UVM and an established epithelioid cell line (Mel 290) to isolate upregulated genes in UM, whereas Smith et al. [25] compared primary tumors from metastatic and non-metastatic patients, thus aiming to identify genes involved in dissemination of metastases.

*SLC16A6* was the most repressed gene in our study. There is little information in the literature on this monocarboxylate transporter, but a recent microarray study identified a repression of this gene in *GNAQ*/*BRAF* mutant UM cells following treatment with selumetinib, a MAPK/ERK kinase (MEK) inhibitor currently in clinical trial for UM [28]. Additional studies are needed to determine its role in UM progression.

It has been proposed that inappropriate activation of stem-cell self-renewal pathways, such as wingless-type MMTV integration site family (WNT) and NOTCH, could lead to the development of cancer [29]. NOTCH and WNT signaling often intersect in stem and progenitor cells and regulate each other transcriptionally. Among the genes shown in Table 3, *CSNK1G1*, *CSNK2A2*, and *CTNNB1* are involved in the WNT pathway, while *ADAM10* and *RBPJ* are regulators of the NOTCH pathway. WNT signaling maintains the integrity of the stem-cell niche and promotes self-renewal by regulating both migration and proliferation [29]. A down-regulation of the cadherin complex could lead to adhesion and morphology abnormalities, which are broadly relevant to tumor progression. Onken et al. [30,31] found previously using microarray a down-regulation of the WNT pathway regulators, including *CSNK2A2* and *CTNNB1*, in metastatic UM. Moreover, the proteolytic processing of DLL1 by ADAM10 is a step that renders this NOTCH ligand inactive [32]. This raises the possibility that ADAM10 is a negative regulator of the NOTCH signaling pathway, which plays an important role in inducing proliferation and invasion in UM [33]. RBPJ is another transcriptional regulator that acts as an activator when bound to the NOTCH intracellular domain (NCID) [34]. Upon NOTCH ligand binding, ADAM-type metalloproteases and γ-secretase containing complex catalyze cleavage of the NOTCH receptor, which leads to the release of the NICD [34]. The NICD migrates to the nucleus where it interacts with RBPJ to recruit a coactivator complex composed of chromatin modifying transcription factors such as histone deacetylases or histone acetylases, resulting in the transcriptional activation of NOTCH target genes [34]. NOTCH target genes often promote cellular differentiation, thus a down-regulation of ADAM10 and RBPJ such as observed in the present study may retain UM cells in an undifferentiated state.

The melanosomes are subcellular organelles that produce the melanin pigment, which is particular to differentiated melanocytes and retinal pigment epithelium. Several genes were previously associated to the pigmentation pathway [35,36], and among them, 6 were found in the present subtracted cDNA library: *DCT*, *RAB27A*, *MITF*, *TRPM1*, *TYR*, and *TYRP1*. A repression of melanin biosynthetic enzymes was observed previously in UM using microarray [17,30]. Also, it was suggested that a decreased expression of MITF in UM cells, such as observed in the present study, might provide a growth advantage by reducing the energy and oxidative stress associated with pigment production [37]. Interestingly, inhibition of MITF in malignant melanoma cells increases the expression of stem cell marker OCT4 [38]. The presence of cancer stem cells (CSCs) in UM has recently emerged as a new hypothesis [16,39]. A subpopulation of highly metastatic UM cells with stem cell properties has been identified previously using the ABCB1 transporter to select the cells [16]. The present observation of a down-regulation of genes involved in pigmentation as well as a positive staining with ABCG2 and OCT4 in UM cells correlate well with an undifferentiated phenotype similar to CSCs.

One limitation of the present study is the use of pooled cDNA for the preparation of the SSH library. Valuable information about the intra-individual variation can be lost when pooled cDNA samples are used. UVM donors and UM patient samples were pooled to get enough starting material, as well as to identify robust gene markers differentially expressed between normal and tumors cells. Nevertheless, SSH findings were validated by microarray using unpooled cDNA from different patients, so we believe the current data are representative of the neoplastic mechanisms leading to tumor development in the great majority of UM patients. Another limitation of the present study is the use of UVM grown in vitro as normal control. Tissue culture conditions could change the gene expression profile of UVM, but when the expression level of pigmentation genes was studied by microarray using cDNA from uncultured choroid (that contains melanocytes and other cell types such as fibroblasts and endothelial cells), pure culture of low-passage UVM, and uncultured primary tumors, both choroid and UVM samples showed higher expression of pigmentation genes than UM tumors (unpublished data).

In summary, the subtracted cDNA library revealed genes that were down-regulated in UM primary tumors. Most these genes had never been reported before. In addition, many of these repressed genes are involved in melanocyte development and differentiation. The race/ethnicity and color of the iris can not explained this difference because all normal donors and UM patients were Caucasians and there was a similar distribution of eyes with light or dark iris in both groups. Deregulation of these pathways support the presence of a subpopulation of cells that exhibit a stem cell-like phenotype in UM. The hypoxic tumor microenvironment has been shown to stimulate specific stem cell signaling pathways and transcription factors, including ABC transporters (such as ABCG2) and OCT4 [40]. Therefore, further studies using low-oxygen conditions may reveal the mechanisms by which hypoxia affects differentiation, tumor growth and metastatic spreading in UM.
